# Predictors of severity and outcome of global developmental delay without definitive etiologic yield: a prospective observational study

**DOI:** 10.1186/1471-2431-14-40

**Published:** 2014-02-12

**Authors:** Loretta Thomaidis, Georgios Zacharias Zantopoulos, Sotirios Fouzas, Lito Mantagou, Chryssa Bakoula, Andreas Konstantopoulos

**Affiliations:** 1 Developmental Assessment Unit, Second Department of Pediatrics, National and Kapodistrian University of Athens School of Medicine, P. & A. Kyriakou Children’s Hospital, Athens, Greece; 2 Department of Pediatrics, University Hospital of Patras, Patras, 265 04 Greece

**Keywords:** Developmental disability, Developmental quotient, Global developmental delay, Intrauterine growth restriction, Prematurity

## Abstract

**Background:**

Although several determinants of global developmental delay (GDD) have been recognized, a significant number of children remain without definitive etiologic diagnosis. The objective of this study was to assess the effect of various prenatal and perinatal factors on the severity and outcome of developmental delay without definitive etiologic yield.

**Methods:**

From March 2008 to February 2010, 142 children with developmental quotient (DQ) <70 and without definitive etiologic diagnosis, were included. Prenatal and perinatal risk factors known to be associated with disordered neonatal brain function were identified. Participants underwent a thorough investigation, an individualized habilitation plan was recommended, and the children were followed-up regularly for a period of 2 < years. The effect of prenatal and perinatal risk factors on the severity and outcome of GDD was assessed by regression analysis.

**Results:**

The mean age at enrolment was 31 ± 12 < months, and the mean DQ 52.2 ± 11.4. Prematurity and intrauterine growth restriction (IUGR) were found to be independently associated with lower DQ values. The mean DQ after the 2-year follow-up was 62.5 ± 12.7, and the DQ difference from the enrollment 10.4 ± 8.9 (median 10; range-10 to 42). DQ improvement (defined as a DQ difference?≥?median) was noted in 52.8% of the children. IUGR, low socio-economic status, and poor compliance to habilitation plan were found to be independently associated with poorer developmental outcomes.

**Conclusions:**

Prematurity and IUGR were found to be significantly and independently related to the severity of GDD in cases without definitive etiologic yield. Poorer 2-year developmental outcome was associated with IUGR, low socioeconomic status and non compliance to habilitation plan. Prematurity was a significant determinant of the outcome only in association with the above mentioned factors.

## Background

Developmental disabilities of childhood represent a group of heterogeneous disorders characterized by age-specific limitations in the acquisition of adaptation and learning skills [1,2]. The term global developmental delay (GDD) is used to describe developmental disability in children less than 5 < years of age [3], and refers to a significant delay in at least two of the major developmental domains: gross and fine motor; speech and language; cognition; social and personal development; and activities of daily living [3,4]. Although it is generally estimated that developmental disabilities affect 5-10% of the pediatric population [1], the reported prevalence varies widely depending on case definition, age range, and population socioeconomic characteristics [5,6]. The precise prevalence of GDD is also unknown. Estimates of 1-5% have been reported in westernized societies [7,8], but the percentage of young children who do not reach their developmental potential is higher in the developing world [6,9].

Accurate determination of the underlying etiology represents an essential step in the management of young children with developmental disabilities [3,4]. However, the reported etiologic yield of GDD is extremely variable, ranging from 10 to 80% in different studies [3,10-12]. Determinants of GDD may be broadly classifiable as prenatal, perinatal and postnatal, and traditionally include genetic syndromes or chromosomal anomalies, hypoxic-ischemic encephalopathy (HIE), cerebral dysgenesis, early severe psychosocial deprivation, antenatal toxin exposure, and central nervous system (CNS) infections [3]. Disordered neonatal brain function, also known as neonatal encephalopathy (NE), is a clinical syndrome associated with life-long chronic disabilities, including cerebral palsy and various cognitive, developmental and behavioral problems [13]. In studies undertaken to assess the underlying etiology of GDD [3,10-12,14,15], NE is often used synonymously with HIE, although the latter actually refers to a sub-set of cases with clear evidence of a perinatal hypoxic-ischemic event [16,17]. However, disordered neonatal brain function is associated with a number of risk factors, including but not limited to perinatal hypoxia-ischemia [13,16-18]. Many of these factors may be preventable or modifiable, and thus, their prompt identification may have significant clinical and prognostic implications [13].

The aim of this study was to assess the effect of various prenatal and perinatal factors known to be associated with NE, on the severity of developmental disability in a cohort of children with significant GDD without definitive etiologic yield. The influence of these factors on the clinical course of GDD was also assessed, taking into account various confounders such as socio-economic status of the family and compliance to the recommended habilitation plan. Our study hypothesis was that factors associated with disordered neonatal brain function may have a significant and independent effect on the severity and course of developmental delay in young children.

## Methods

The study was conducted between March 2008 and February 2012 at the Developmental Assessment Unit of the “P amp; A Kyriakou” Childrens Hospital in Athens, Greece, one of the main referral Developmental Pediatric centers of the country.

### Participants and protocol

During a 2-year inclusive period (March 2008–February 2010), children younger than 5 < years of age referred to our Unit for suspected developmental delay were screened for eligibility. Participants should have had significant GDD, (defined as a developmental quotient–DQ <70) without a definitive etiologic diagnosis including HIE, pathologic neonatal neuroimaging, chromosomal abnormalities, genetic syndromes, metabolic or neuromuscular disorders, congenital infections, central nervous system (CNS) malformations, congenital hypothyroidism, CNS infections or severe injuries beyond the neonatal period, toxin exposure, or severe psychosocial neglect. If such a disorder was diagnosed at presentation (or during the following 2-year follow-up period), the child was not included in the study (or was, respectively, excluded). Children with autistic features were also excluded.

Developmental assessment was completed by an experienced developmental pediatrician using the Bayley Scales of Infant Development [19] for children up to 3 < years of age, and the Griffiths Scales for Mental Development [20] and the Athina Test [21] for older children. The lower DQ value was taken into account when both Griffiths Scales for Mental Development and the Athina Test were used. Developmental quotient was calculated as percentage of functional age compared to chronological age.

Information regarding the perinatal and the neonatal period was obtained by a systematic examination of the participants’ medical records, including personal health booklets, hospital discharge notes, follow-up or outpatient information notes, and any related laboratory or imagistic examination from the personal medical files. A detailed history was also obtained from the parents and the referring physician was contacted for any additional information. The diagnosis of HIE was based on the criteria established by the American Academy of Pediatrics task Force on Neonatal Encephalopathy and Cerebral Palsy [17], which include evidence of metabolic acidosis in fetal umbilical cord arterial blood at delivery, early onset of severe or moderate neonatal encephalopathy, and cerebral palsy of the spastic quadriplegic or dyskinetic type. If the diagnosis of HIE was not definitive, but there was evidence of a non-specific asphyxial insult (i.e., a sentinel hypoxic event occurring immediately before or during labor, sudden and sustained fetal bradycardia or absence of fetal heart rate variability, Apgar score of less than 5 beyond 5 < minutes) [16] without pathologic findings in neonatal neuroimaging, the child was considered as having experienced birth asphyxia without significant CNS involvement and was not excluded from the study. Pathologic neonatal neuroimaging (brain ultrasound, CT, MRI) findings included intraventricular hemorrhage, ventriculomegaly (symmetrical or asymmetrical), periventricular hyperechogenicity (localized or diffused), multiple micro or macro cysts in the periventricular white matter, and cortical or subcortical infarcts. Children with such findings were excluded from the study irrespectively of the clinical diagnosis.

Factors known to be associated with NE [13,16-18] were also recorded. These included *in vitro* fertilization (IVF), alcohol, illicit drugs, and tobacco smoke exposure during pregnancy, multiple gestation, prematurity (defined as gestational age <37 < weeks), post-term birth (defined as gestational age >42 < weeks), intrauterine growth restriction (IUGR–defined as a ponderal index <10th percentile), hypertensive disease of pregnancy (preeclampsia, eclampsia), hemorrhagic complications of pregnancy (placental abruption, placenta previa), mode of delivery, birth asphyxia (defined by the criteria analyzed above), neonatal hypoxia (need for ventilatory support due to significant respiratory deterioration, including respiratory distress syndrome and apneas), neonatal infection, and severe hyperbilirubinemia. Failure to thrive was defined as child’s weight for age below the fifth percentile of the standard Greek growth charts [22]. The socio-economic status of the family was also assessed based on parental profession (i.e., manual, employee, or academic) and years of education, as was described elsewhere [23].

Children underwent a thorough investigation according to the guidelines applied in our Department, including biochemistry, metabolic tests, chromosome analysis, cytogenic study, electroencephalography (EEG), and neuroimaging (CT, MRI). A habilitation plan (special education support, language therapy, behavioral or occupational therapy, and social support in the community) was recommended on an individualized case-by-case basis. Participants were asked to attend our Department regularly at 6-months intervals. Only follow-up data obtained during the first 2 < years after the initial presentation were used in the present study. At each follow-up visit, progress (in terms of DQ improvement) was assessed and investigations and treatment plans were reviewed.

The present study was strictly adhered to the protocols for the management of GDD applied in our institution. Physical examination, specific laboratory testing, and treatment options and recommendations were carried out only at the discretion of the attending specialist on an individualized case-by-case basis. The study was approved by the Ethics Committee of the “P amp; A Kyriakou” Childrens Hospital. An informed consent was obtained from one of the parent of each child at the enrollment.

### Statistical analysis

All data were recorded on a standardized data sheet. Descriptive statistics were generated and exploratory analysis on the relation between DQ at the enrollment and the presence of risk factors was performed. Linear regression analysis was used to assess both crude (unadjusted) and combined (adjusted) effects of these factors on the initial GDD severity. The DQ difference between the 2-year visit and the initial assessment was calculated and DQ improvement was defined as a DQ difference equal or greater than the median DQ difference of the study cohort. Logistic regression analysis was used to assess the crude and combined effects of risk factors on DQ improvement (GDD course) taking also into account confounders such as lower socioeconomic status and non adherence to habilitation plan. These effects were presented as risk ratios (RR). A significance level of 0.05 was selected for all hypotheses. Statistical analysis was performed using SPSS v.17 (SPSS Inc., Chicago, IL).

## Results

During the inclusive period, a total of 296 children younger than 5 < years of age were referred for suspected developmental delay to our Developmental Pediatric Department and were consecutively screened for enrolment in the study. Of these, 21 did not meet the criteria for significant GDD upon specialty evaluation, 7 were diagnosed with autism, 79 children were excluded due to a definitive etiologic diagnosis (HIE 28, pathologic neonatal neuroimaging 21, chromosomal abnormalities 11, genetic syndromes 9. metabolic diseases 3, neuromuscular disorders 3, CNS infections beyond the neonatal period 2, and CNS injury 2), 6 children suffered severe psychosocial deprivation, 29 were excluded due to incomplete personal medical records, and 12 were lost to follow-up. The remaining 142 children constituted our main study cohort.

Ninety of the participants (63.4%) were males. The mean age at the enrolment was 31 ± 12 < months. Sixty children (42.2%) were from families of low socio-economic status. Failure to thrive was documented in 23 children (16.2%). The mean DQ at the enrollment was 52.2 ± 11.4. The DQ of children initially assessed by the Bayley Scales of Infant Development (N?=?88) was 51.7 ± 10.9, whereas of those assessed by Griffiths Scales for Mental Development and Athina Test 53 ± 13.5 (P?=?0.530). Lower socio-economic status and failure to thrive were both associated with significantly lower DQ values (44.3 ± 12.1 *vs.* 58.1 ± 11.7; *P* <0.001 and 43.9 ± 11.8 *vs.* 53.8 ± 11.1; *P* <0.001 respectively).

On exploratory analysis, factors associated with lower DQ were prematurity, multiple gestation, IUGR, hypertensive disease of pregnancy, birth asphyxia, and neonatal hypoxia (Table < 1). Unadjusted linear regression analysis confirmed the above findings (Table < 2). When the combined effect of these factors was assessed, only prematurity and IUGR were found to be independently associated with significantly lower DQ (Table < 2).

**Table 1:** Initial developmental quotient in relation to various prenatal and perinatal risk factors

		**N**	**DQ**	***P***
***Prenatal factors***				
Fecundation *in vitro*	yes	7	46 ± 12.1	0.124
	no	135	52.5 ± 10.8	
Drug exposure in pregnancy*	yes	14	51.2 ± 11.3	0.734
	no	128	52.3 ± 11.5	
Tobacco smoke exposure in pregnancy	yes	42	50.0 ± 11.4	0.127
	no	100	53.1 ± 10.8	
Multiple gestation	yes	12	44.1 ± 11.2	0.012
	no	130	52.9 ± 11.4	
Prematurity	yes	43	46.8 ± 11.6	<0.001
	no	99	54.5 ± 11.0	
Post-term birth	yes	11	50.1 ± 12.3	0.517
	no	131	52.4 ± 11.2	
IUGR	yes	32	42.8 ± 11.1	<0.001
	no	110	54.9 ± 11.4	
Hypertensive disease of pregnancy †	yes	9	44.2 ± 10.9	0.030
	no	133	52.7 ± 11.3	
Hemorrhagic complications of pregnancy ‡	yes	13	48.3 ± 11.6	0.180
	no	129	52.6 ± 10.9	
***Perinatal factors***				
Cesarean section	yes	55	51.3 ± 11.3	0.445
	no	87	52.8 ± 11.4	
Birth asphyxia	yes	18	45.8 ± 12.0	0.012
	no	124	53.1 ± 11.3	
Neonatal hypoxia §	yes	40	48.4 ± 11.5	0.014
	no	102	53.7 ± 11.4	
Neonatal infection	yes	9	46.3 ± 12.2	0.109
	no	133	52.6 ± 11.3	

Data are means ± SD.

*exposure to alcohol and illicit drugs; † preeclampsia, eclampsia; ‡ placental abruption, placenta previa; § need for ventilatory support due to respiratory distress or severe apneas;

DQ indicates developmental quotient; IUGR intrauterine growth restriction.

**Table 2:** Effect of various prenatal and perinatal risk factors on the severity of developmental delay

	**Unadjusted effect**	**Adjusted effect**
***Prenatal factors***		
Fecundation *in vitro*	-0.145	-0.101
Drug exposure in pregnancy§	-0.021	-0.019
Tobacco smoke exposure in pregnancy	-0.147	-0.113
Multiple gestation	-0.193*	-0.098
Prematurity	-0.276*	-0.177†
Post-term birth	-0.059	-0.032
IUGR	-0.301*	-0.256*
Hypertensive disease of pregnancy ||	-0.160‡	-0.128
Hemorrhagic complications of pregnancy ¶	-0.150	-0.106
***Perinatal factors***		
Cesarean section	-0.082	-0.049
Birth asphyxia	-0.190*	-0.125
Neonatal hypoxia**	-0.181†	-0.090
Neonatal infection	-0.150	-0.089

Data are standardized coefficients *beta* resulted by linear regression analysis. Unadjusted effect is the effect of each factor separately. Adjusted effect is the effect of each factor adjusted for the effect of the others and for socio-economic status (multiple regression model).

*P <0.001; † P <0.01; ‡ P <0.05.

§ exposure to alcohol and illicit drugs; || preeclampsia, eclampsia; ¶ placental abruption, placenta previa; **need for ventilatory support due to respiratory distress or severe apneas;

IUGR indicates intrauterine growth restriction.

During the 2-year follow-up period, all children underwent a thorough developmental investigation, including biochemistry and endocrinological investigations (N?=?142), metabolic tests (N?=?59), chromosome analysis (N?=?138), cytogenic studies (N?=?46), EEG (N?=?77) and neuroimaging (N?=?142). The recommended habilitation plan included (on individual basis) special education support (N?=?131), language therapy (N?=?97), behavioral therapy (N?=?20), occupational therapy (N?=?89), and social support in the community (N?=?98). Compliance to the above recommendations was noted in 92 cases (64.8%). One hundred children (70.5%) had 4 follow-up visits, 34 (23.9%) 3 visits, and 8 (5.6%) 2 visits. All children presented to the follow-up visit at 2 < years after the enrolment.

The mean DQ at the 2-year visit was 62.5 ± 12.7, and the DQ difference between 2-years and enrolment 10.4 ± 8.9 (median 10; range-10 to 42). The DQ difference in children initially assessed by the Bayley Scales of Infant Development and later by Griffiths Scales for Mental Development and Athina Test (N?=?88) was 9.7 ± 10.8, whereas that of children assessed by Griffiths Scales for Mental Development and Athina Test in both instances (N?=?54) 11.5 ± 10.1 (P?=?0.325). DQ improvement was defined as a DQ difference ≥10 (population median), and was noted in 75 children (52.8%); 4 participants showed further DQ deterioration. Prematurity, IUGR, neonatal hypoxia, low socio-economic status, and compliance to treatment were associated with lower DQ differences (Table < 3). IUGR, low socio-economic status, and compliance to habilitation plan were found to be independently associated with poorer GDD outcomes (Table < 4).

**Table 3:** Course of developmental delay over the 2-year surveillance period in relation to various factors

**Factors**		DQ difference*		***P***
		**Median**	**Range**	
***Prenatal***				
Fecundation *in vitro*	yes	9.2	-4.0 to 23.0	0.687
	no	10.0	-10 to 42.0	
Drug exposure in pregnancy †	yes	8.5	-10 to 27.0	0.244
	no	10.0	-5.0 to 42.0	
Tobacco smoke exposure in pregnancy	yes	9	-10.0 to 35.0	0.340
	no	10.0	-5.0 to 42.0	
Multiple gestation	yes	9.0	-10.0 to 20.0	0.169
	no	10.0	-5.0 to 42.0	
Prematurity	yes	7.5	-10.0 to 18.0	0.022
	no	10.0	-4.0 to 42.0	
IUGR	yes	7.0	-10.0 to 15.0	0.006
	no	10.0	0.0 to 42.0	
Hypertensive disease of pregnancy ‡	yes	9.5	-10.0 to 38.0	0.904
	no	10.0	-5.0 to 42.0	
Hemorrhagic complications of pregnancy §	yes	9.0	-10.0 to 36.0	0.820
	no	10.00	-5.0 to 42.0	
***Perinatal***				
Cesarean section	yes	10.0	-10.0 to 36.0	0.880
	no	10.0	-5.0 to 42.0	
Birth asphyxia	yes	9.0	-5.0 to 35.0	0.620
	no	10.0	-10.0 to 42.0	
Neonatal hypoxia ||	yes	8.0	-10.0 to 15.0	0.024
	no	10.5	0.0 to 42.0	
Neonatal infection	yes	9.0	-4.0 to 42.0	0.870
	no	10.0	-10.0 to 35.0	
***Other***				
Low socio-economic status	yes	6.0	-10.0 to 10.0	<0.001
	no	11.0	0.0 to 42.0	
Adherence to recommendations and treatment	yes	12.0	0.0 to 42	<0.001
	no	5.0	-10.0 to 12	

*calculated as DQ_(2-year visit)_–DQ_(initial visit)_.

Between-group differences are calculated with Mann–Whitney U test.

†exposure to alcohol and illicit drugs; ‡ preeclampsia, eclampsia; § placental abruption, placenta previa; || need for ventilatory support due to respiratory distress or severe apneas.

DQ indicates developmental quotient; IUGR intrauterine growth restriction.

**Table 4:** Effect of various factors on the course of developmental delay

**Factors**	**Risk ratio for poor DQ progress † (95% CI)**	
	**Unadjusted**	**Adjusted**
***Prenatal***		
Fecundation *in vitro*	1.3 (0.3–7.9)	1.1 (0.3–6.5)
Drug exposure in pregnancy ‡	1.3 (0.5–6.6)	1.4 (0.6–7.4)
Tobacco smoke exposure in pregnancy	1.1 (0.7–2.2)	1.2 (0.7–3.0)
Multiple gestation	1.5 (0.8–6.9)	1.6 (0.8–7.4)
Prematurity	3.0 (1.3–7.8)*	2.1 (0.8–7.2)
IUGR	4.3 (1.5–9.0)*	5.1 (1.8–11.0)*
Hypertensive disease of pregnancy §	1.1 (0.3–7.8)	1.0 (0.3–6.9)
Hemorrhagic complications of pregnancy ||	1.2 (0.4–7.1)	1.0 (0.4–6.5)
***Perinatal***		
Cesarean section	1.0 (0.5–2.8)	1.0 (0.5–3.2)
Birth asphyxia	1.5 (0.7–7.7)	1.6 (0.8–8.1)
Neonatal hypoxia ¶	2.3 (1.2–7.7)*	2.0 (0.8–7.0)
Neonatal infection	1.2 (0.4–9.0)	1.2 (0.5–8.5)
***Other***		
Low socio-economic status**	4.8 (2.4–9.9)*	4.6 (2.0–7.8)*
No adherence to treatment ††	5.5 (2.9–10.4)*	8.5 (3.4–15.5)*

Data are relative risk ratios calculated by logistic regression analysis. Unadjusted effect is the effect of each factor separately. Adjusted effect is the effect of each factor adjusted for the effect of the others (multiple regression model). Significant effect is indicated by (*).

† Poor DQ progress defined as DQ_(2-year visit)_–DQ_(initial visit)_ less than population median.

‡ exposure to alcohol and illicit drugs; § preeclampsia, eclampsia; || placental abruption, placenta previa; ¶ need for ventilatory support due to respiratory distress or severe apneas.

**N?=?60; †† N?=?50.

DQ indicates developmental quotient; IUGR intrauterine growth restriction.

## Discussion

Developmental disabilities represent a common problem in pediatric care and a common reason for referral to developmental or neurology subspecialists [7,8]. However, their heterogeneous nature, together with the wide-ranging underlying etiology and the considerable uncertainty with respect to the extent of investigations to be undertaken, poses a particular challenge for the individual practitioner [3]. Thus, although several prenatal, perinatal and postnatal determinants of early developmental disability have been recognized, a significant number of children with GDD remain without a definitive etiologic diagnosis [3,10-12]. Neonatal encephalopathy represents one of the main conditions which result in serious limitations in the acquisition of adaptation and learning skills. Given its estimated incidence of 2.0 to 6.0 per 1000 live births [13], this syndrome is apparently responsible for a significant proportion of GDD. Neonatal encephalopathy is associated with a number of adverse prenatal or perinatal events [16-18], but what remains comparatively unclear to date, is the independent contribution of these factors on the severity and clinical course of developmental delay. To the best of our knowledge, the present study is the first to assess this effect in a cohort of children with significant GDD without definitive etiologic yield.

Factors known to be associated with disordered neonatal brain function, such as prematurity, multiple gestation, IUGR, hypertensive disease of pregnancy, birth asphyxia, and neonatal hypoxia, were all associated with lower DQ in our study. However, only prematurity and IUGR were independently associated with more severe developmental delay, suggesting that these were the most important predictors of the severity of GDD in our cohort. On the other hand, prematurity, IUGR, and neonatal hypoxia, were also related to a poorer 2-year developmental outcome. However, when their combined effect was assessed taking also into account the significant effect of lower socioeconomic status and non adherence to treatment, only IUGR was found to be an independent predictor of the GDD clinical course.

Accumulate evidence suggests that prematurity is not only associated with interruption of brain development at early and critical neurodevelopmental stages, but it also perturbs the trajectory of normal cerebral development after birth [24]. Therefore, children born preterm, and especially those who survive severe or extreme prematurity, will face a number of neurobehavioral, adaptive and social challenges. Interestingly, these neuromotor, cognitive and behavioral impairments seems to be present across the whole preterm spectrum, from extreme prematurity to late-preterm birth [24,25]. Although in the present study prematurity was an important and independent predictor of the severity of GDD, its negative effects on the acquisition of developmental skills seems to be modifiable. Indeed, prematurity was not an independent predictor of poorer GDD outcome in our cohort, as long as it was not associated with low socio-economic status and poor compliance to habilitation plan. Although our findings need further elucidation (prospective studies, larger sample sizes, different populations and settings), they underscore the importance of early developmentally supportive intervention to ensure more optimal neurocognitive outcomes among children born preterm [26].

On the other hand, the developing brain may be more vulnerable than previously thought to chronic intrauterine substrate deprivation [27]. Recent evidence from primate animal models suggest that even moderate maternal undernutrition may lead to major disturbances in the architecture and maturation of the developing cortical neuronal network with a potential impact on brain function over the lifespan [28]. Moreover, data supporting a relation between maternal undernutrition during pregnancy and cognitive impairment in children have been also reported [29]. In agreement with this report, IUGR was proven to be a major determinant of severity and poor outcome of GDD in our study. This effect was independent of other negative perinatal and socio-environmental risk factors, thus providing further support to a possible link between IUGR and developmental disabilities in children.

Abnormalities in neuroimaging during the neonatal period have been considered as a major predictor of cognitive disability in childhood [24,30,31], and it has been shown that even mild or subclinical findings may be associated with neurocognitive impairment in later life [30,32]. Since in our study children with abnormal neonatal neuroimaging were excluded, conclusions on this association cannot be drawn. However, our results clearly suggest that in the face of a young child with developmental disability without etiologic yield, the presence of specific perinatal risk actors should be carefully assessed irrespectively of the reassuring results of neonatal neuroimaging.

Our study has some limitations. First, although participants were included prospectively and were followed-up for a period of 2 < years, data on prenatal and perinatal risk factors were collected retrospectively from a detailed history and the personal medical records. Children with incomplete medical records were excluded from the study in order to minimize any related bias, but incomplete data assessment still remains a possibility. Second, the severity and the 2-year outcome of GDD were defined based exclusively on DQ values. Although all DQ determinations were made by the same experienced developmental pediatrician, a possible bias due to intraobserver variability cannot be excluded. To minimize the effects of such a bias, we chose to include only children with significant developmental disability (i.e., those with DQ <70) and to define DQ improvement based on the median 2-year DQ difference which represented an intrinsic characteristic of our study cohort. Third, specific laboratory testing was carried out on an individualized case-by-case basis at the discretion of the attending specialist, therefore, a complete diagnostic panel was not ordered in all participants. However, the present study was not designed to assess the underlying etiology of GDD in our cohort. Finally, since the sample population comes from a single referral center in Greece, our results may not be generalized to other populations and settings where socioenvironmental factors, referral patterns, subspecialty assessment protocols, and accessibility to medical care and developmental supportive interventions may be different.

## Conclusion

In conclusion, the results of the present study indicate that specific prenatal and perinatal factors related to disordered neonatal brain function, such as prematurity and IUGR, may be significant and independent predictors of the severity of GDD in cases without definitive etiologic yield. In contrast to IUGR, however, prematurity was not a significant determinant of poorer developmental outcome, provided that it was not associated with low socioeconomic status or poor compliance to habilitation plan. Our findings suggest that these risk factors may be preventable or modifiable, and thus, their prompt identification combined with an early supportive intervention strategy may have significant implications on the long-term outcome of developmental disability.

## Abbreviations

GDD: Global developmental delay; HIE: Hypoxic-ischemic encephalopathy; CNS: Central nervous system; NE: Neonatal encephalopathy; DQ: Developmental quotient; IVF: *In vitro* fertilization; IUGR: intrauterine growth restriction.

## Competing interests

The authors declare that they have no competing interests.

## Authors’ contributions

LT: Dr. T conceptualized and designed the study, drafted the initial manuscript, and approved the final manuscript as submitted. SF: Dr. F carried out the analyses, drafted the initial manuscript, and approved the final version as submitted. GZZ and LM: Dr. Z and Dr. M coordinated and supervised data collection, critically reviewed the manuscript, and approved the final manuscript as submitted. CB and AK: Prof. B and Prof. K conceptualized and designed the study, critically reviewed the manuscript, and approved the final version as submitted.

## Pre-publication history

The pre-publication history for this paper can be accessed here:

http://www.biomedcentral.com/1471-2431/14/40/prepub
